# Therapeutic Potential of Glucagon-like Peptide-1 Agonists in Polycystic Ovary Syndrome: From Current Clinical Evidence to Future Perspectives

**DOI:** 10.3390/biomedicines10081989

**Published:** 2022-08-16

**Authors:** Mojca Jensterle, Rok Herman, Andrej Janež

**Affiliations:** 1 Department of Endocrinology, Diabetes and Metabolic Diseases, University Medical Center Ljubljana, 1000 Ljubljana, Slovenia; 2 Department of Internal Medicine, Faculty of Medicine, University of Ljubljana, 1000 Ljubljana, Slovenia

**Keywords:** GLP-1 receptor agonists, incretins, polycystic ovary syndrome, weight management, high metabolic risk, guidelines, future perspective

## Abstract

Despite the continuous effort to understand the pathophysiology and determine potential therapeutic targets, PCOS treatment largely depends on lifestyle intervention and symptomatic management of individual signs and symptoms. International guidelines recognize the importance of weight reduction as a cornerstone for the achievement of better metabolic, reproductive, and cardiovascular outcomes in PCOS women who are overweight or obese. With its profound weight loss potential in patients with or without diabetes, the administration of GLP-1 receptor agonists has been investigated in overweight/obese women with PCOS in several single-center randomized control trials with considerable variation in the dosing regimen, follow-up duration, and outcome measurements over recent years. Most trials reported superior weight loss effects of GLP-1 receptor agonists compared to lifestyle changes or metformin, with additional metabolic, reproductive, and cardiovascular benefits in this population. However, their use is currently not widely accepted by the clinical community that treats this population. The major concern is how to balance the reproductive and metabolic treatment strategies since the use of GLP-1 receptor agonists requires effective contraception while on therapy and a washout period before pregnancy. Both approaches are not mutually exclusive, yet the best choice requires a careful assessment of the clinical context. Knowing a patient’s individual circumstances, precise clinical sub-phenotyping, and regular monitoring are crucial components for the safe and effective use of these new tools. In the present narrative review, we explore the current clinical evidence and provide the future perspectives and challenges for their implementation in PCOS management.

## 1. Introduction

Polycystic ovary syndrome (PCOS) is the most common metabolic and hormonal disorder in reproduction-aged women [1]. Its pathogenesis involves multiple organ systems and is tightly associated with a higher predisposition and prevalence of abdominal obesity and insulin resistance. Patients present with various reproduction disorders such as hyperandrogenemia, imbalanced gonadotropin secretion, ovulatory dysfunction, polycystic ovarian morphology, infertility, miscarriages, premature birth, and gestational diabetes. In addition, dysglycemia, fatty liver disease, obstructive sleep apnea, increased risk markers for cardiovascular disease, depression, disordered eating, and sexual dysfunction have been recognized as frequent co-morbidities of this syndrome [2].

In recent years, intense research has been aimed at understanding the pathophysiology and therapeutic targets for this disorder and its metabolic and reproductive consequences, with insulin resistance, androgen biosynthesis, and neuroendocrine core pathways being recognized as the main drivers of the disease [3]. However, despite decades of investigative effort and significant progress in subtyping the genetic and clinical phenotypes of this heterogeneous syndrome, the etiology of PCOS remains unclear, and its treatment mainly symptomatic.

Weight loss has health benefits for all overweight and obese PCOS patients regardless of the dominant pathophysiological pathway and clinical phenotype. At present, lifestyle change that is centered on weight reduction, combined with symptomatic treatment, is dominant. However, women with PCOS face several hurdles to successful lifestyle change, such as reduced metabolic flexibility, lack of intrinsic motivation, negative body image, depression, eating disorders, and altered risk perception [4,5]. Linking PCOS to inherited insulin resistance that is frequently aggravated by obesity-induced insulin resistance paved the way for trials of diabetes drugs in PCOS [6,7]. With encouraging early studies, metformin gained an essential role in the management of PCOS with effects that were comparable to lifestyle modification and the superior effects that were achieved by the combination of both approaches [8]. Although the combination of metformin and lifestyle intervention has been shown to reduce weight by up to 5% and improve insulin resistance, hyperandrogenism, menstrual function, and fertility in this population [9], the effects of metformin in combination with lifestyle modification are considered modest and compromised by low adherence [8].

The recent developments in weight loss pharmacotherapy have transpired the advent of a new paradigm in weight management across multiple conditions [10]. The use of a prespecified weight loss amount as a primary biomarker is increasingly being used in patients with adipose-based chronic diseases (ABCD) [10]. The metabolic phenotype of PCOS, central adiposity, abnormalities in adipose tissue function and distribution, dysglycemia, hyperlipidemia, non-alcoholic fatty liver disease, acanthosis nigricans, and unfavorable risk profile for cardiovascular diseases all represent features of ABCD [10,11,12].

While any amount of weight loss is effective for treating and preventing ABCD complications, there is a clear weight loss dose-response in achieving clinical benefit. [10]. Based on the current data on the weight loss that is needed to alleviate manifestations of other conditions such as prediabetes, diabetes, obstructive sleep apnea, non-alcoholic fatty liver disease, and the prevention of cardiovascular events and mortality, interventions that are able to produce between 10% and 20% weight loss are required [10]. Lifestyle interventions and metformin are insufficient in enabling such a treat-to-target approach in the metabolic phenotype of PCOS.

Profound weight loss effects in diabetic and non-diabetic patients gave birth to the idea that GLP-1 receptor agonists (GLP-1RAs) could be used in a subgroup of women with PCOS. Thus, several studies have been conducted over the past years with the aim of testing the efficacy and safety of GLP-1RAs in PCOS management. In addition, a lot of effort has been put into comparing the effectiveness of metformin, GLP-1RAs, and their combination in the management of mainly metabolic PCOS phenotype [13]. Despite the availability of several GLP-1RAs, exenatide and liraglutide are the only two GLP-1RAs that have been systematically studied so far. The first results with the most potent long-acting GLP-1RA for the treatment of obesity, subcutaneous (s.c) semaglutide, that safely produces ≥10% of placebo-subtracted weight loss and ≥15% weight loss in more than half of the treated patients [10], have been reported only in one pilot study with a PCOS population [14].

In this narrative review, we will update and review the evidence for the clinical applicability of GLP-1RAs in PCOS patients, especially when managing the metabolic and reproductive consequences of this disorder. In addition, we will briefly discuss the potential function of GLP-1 agonism in PCOS and the regulation of the hypothalamic-pituitary-gonadal (HPG) axis, which might hypothetically go beyond mere weight reduction. In the end, we will provide our view of the current position and future perspectives of GLP-1RAs in the management of PCOS based on the literature data and the extensive clinical and research experiences of our group.

## 2. Current Position of GLP-1 Receptor Agonists in PCOS Management

The use of GLP-1RAs in PCOS is currently not widely accepted and recognized by clinicians. The 2018 international guidelines for the management of PCOS highlight that excess weight is a vital concern for women with PCOS and that anti-obesity medications (AOM) could be a viable option for the treatment of obesity in PCOS after and in combination with lifestyle intervention. However, their specific role in PCOS and reproductive-aged women remains unclear [15].

In the following chapters, we will provide an updated review of the clinical efficiency of GLP-1RAs in alleviating various aspects of this disorder to offer an opportunity to reconsider and potentially expand the current treatment options with a powerful pharmacological agent that provides superior results compared to lifestyle changes or metformin in metabolic aspects of the syndrome.

### 2.1. The Efficacy of GLP-1 Receptor Agonists in Weight Management in PCOS

In 2014, the United States Food and Drug Administration (FDA) approved liraglutide as the first GLP-1RA that can be used for obesity management in patients without diabetes [16]. In 2021, semaglutide followed with indications in obese patients (BMI above 30 kg/m^2^) or overweight patients (BMI above 27 kg/m^2^) with at least one ABCD [17]. GLP-1RAs alone or combined with metformin have been investigated in several small studies with overweight/obese PCOS women [18]. Table 1 summarizes the clinical studies that measured the weight-reducing effects of GLP-1RAs in PCOS patients as one of their outcomes.

Additional insight was provided by a network meta-analysis including 23 studies and 951 women, which compared the effectiveness of liraglutide, orlistat, and metformin in promoting weight loss in PCOS women. Liraglutide monotherapy was superior in reducing body weight and waist circumference. Furthermore, its efficacy was the highest at the daily dose of 3 mg [19].

**Table 1:** Clinical studies that measured the weight loss effects of GLP-1RAs in PCOS.

Population Studied	Study Type	Duration	Study Arms	Weight Loss	Other Remarks	Ref
40 obese nondiabetic women with PCOS who had lost <5% body weight during pretreatment with metformin	Open-label, prospective study	12 weeks	Metformin 1000 mg BID	−1.2 ± 1.4 kg	WC also decreased by 5.5 ± 3.8 cm in the combination arm compared with 3.2 ± 2.9 cm in liraglutide and 1.6 ± 2.9 cm in the metformin arm. The majority of patients who achieved at least 5% of weight reduction were on combination therapy or liraglutide monotherapy.	[20]
			liraglutide 1.2 mg QD s.c.	−3.8 ± 3.7 kg		
			metformin 1000 mg BID and liraglutide 1.2 mg QD s.c	−6.5 ± 2.8 kg		
32 obese women with newly diagnosed PCOS	Open-label, prospective study	12 weeks	Metformin 1000 mg BID	−2.3 kg	Comparable results were found for the reduction of BMI, WC and whole-body fat mass. However, in a subgroup of patients with the combination of extreme obesity and insulin resistance, the patients achieved better results with liraglutide compared to metformin.	[21]
			Liraglutide 1.2 mg QD s.c.	−3.0 kg		
84 overweight/obese women with PCOS	Observational study	a minumum of 4 weeks; a mean duration of treatment was 27.8 weeks	Starting dose was 0.6 mg liraglutide given s.c. QD. If the weight was not reduced, the dose was increased to 1.2 mg and if necessary to 1.8 mg.	−9.0 kg	81.7% of patients achieved beyond 5% weight loss, and 32.9% of patients achieved more than 10% weight loss.	[22]
72 women with PCOS, with a BMI > 25 kg/m^2^ and/or insulin resistance	Prospective, double-blind, placebo-controlled, randomized clinical trial	26 weeks	Placebo	0.2 kg	Body weight reduction of more than 5% was achieved in 55% and 14% of participants in the liraglutide and placebo groups, respectively. In addition to liver fat content, VAT and SAT were reduced by 18.6% and 10.0%, respectively.	[23]
			liraglutide 1.8 mg QD s.c	−5.2 kg		
44 obese women with PCOS	Open-label, prospective, randomized control trial	12 weeks	Liraglutide 1.2 mg QD s.c.	−3.8 ± 3.5 kg	59.1% of patients in the cobination groups vs. 42.9% of patients in the liraglutide-only group achieved beyond 5% weight reduction.	[24]
			metformin 1000 mg BID and liraglutide 1.2 mg QD s.c.	−6.2 ± 2.4 kg		
31 obese patients with PCOS	Retrospective study	6 months	Metformin 500 or 1000 mg daily	−4.9 kg	Liraglutide was superior in the analysis of the number of patients that achieved 5% or 10% weight loss.	[25]
			Liraglutide doses of 1.8 mg and 3.0 mg or semaglutide dosing up to 1 mg	−9.1 kg		
50 overweight/obese PCOS women	Open-label prospective, randomized, clinical trial	12 weeks	Metformin 500 mg TID	−2.1 ± 3.0 kg	WC decreased by 4.63 ± 4.4 cm in combination group compared with 1.72 ± 3.07 cm in the metformin-only group.	[26]
			metformin 500 mg TID, exenatide 2 mg QW	−3.8 ± 2.4 kg		
60 overweight oligoovulatory women with PCOS	Open-label prospective, randomized, clinical trial	24 weeks	Metformin 1000 mg BID	−1.6 ± 0.2 kg	Combination therapy was more efficient compared to to exenatide or metformin in reducing abdominal fat.	[27]
			exenatide 10 mcg BID	−3.2 ± 0.1 kg		
			metformin 1000 mg BID and exenatide 10 mcg BID	−6.0 ± 0.5 kg		
19 obese women with PCOS	Open label, prospective	6 months	Liraglutide 1.8 mg QD	−3.0 ± 4.2 kg	/	[28]
45 obese PCOS women	Open-label, prospective, randomized clinical trial	12 weeks	Metformin 1000 mg BID	−0.2 ± 1.8 kg	Liraglutide also resulted in significant decrease in VAT area and was superior in reducing WC.	[29]
			roflumilast 500 mcg QD	−2.1 ± 2.0 kg		
			liraglutide 1.2 mg QD	−3.1 ± 3.5 kg		
30 obese PCOS women	Open-label prospective randomized clinical trial	12 weeks	Metformin 1000 mg BID and liraglutide 1.2 mg QD	−3.6 ± 2.5 kg	WC reduction in liraglutide arm was greater than in combination.	[30]
			liraglutide 3.0 mg QD	−6.3 ± 3.7 kg		
28 infertile obese PCOS patients	Open-label prospective randomized clinical trial	12 weeks	Metformin 1000 mg BID	−7.0 ± 6.0 kg	Weight reduction beyond 5% was seen in 69.2% of patients in the combination group and 57.1% of patients in the metformin-only group. Significant and similar decreases in WC, VAT area, and volume were noticed between groups.	[31]
			metformin 1000 mg BID combined with liraglutide 1.2 mg QD	−7.5 ± 3.9 kg		
176 overweight/obese women with PCOS	Open-label prospective, randomized clinical trial	24 weeks	Metformin 1000 mg BID	−2.3 ± 0.6 kg	47% of patients achieved beyond 5% weight loss with exenatide therapy in the first 12 weeks, but no subject demonstrated similar weight loss with MET therapy. The decrease in WC was more significant in patients on exenatide than those in patients on metformin. Exenatide therapy resulted in significant decreases in abdominal fat.	[32]
			exenatide 10 μg BID (first 12 weeks), metformin 1000 mg BID (second 12 weeks)	−4.3 ± 1.3 kg		
30 overweight/obese anovulatory women with all 3 Rotterdam criteria	Open label, prospective study	16 weeks	exenatide 5 mcg BD for 4 weeks then 10 mcg BD for 12 weeks	−3.2 kg	There was no effect on WC but there was a reduction in hip circumference.	[33]
32 overweight/obese PCOS patients	Prospective study	12 weeks	the initial dose of exenatide 5 μg BD was increased to 10 μg BD after 1 month	−6.0 kg	After exenatide treatment, the body adipose distribution—related indexes, including body fat content, WC, and hipline circumference, decreased.	[34]
119 nondiabetic obese women with PCOS	Single-blinded, randomized controlled trial	24 weeks	once-weekly 2 mg exenatide (EQW)	−4.1 kg	The combination of exenatide and dapagliflozin resulted in superior weight and total body fat reductions than either therapy individually.	[35]
			dapagliflozin 10 mg daily (DAPA)	−1.4 kg		
			coadministered EQW/DAPA	−6.0 kg		
			DAPA/extended-release (ER) metformin 2000 mg daily (DAPA/MET)	−1.8 kg		
			phentermine 7.5 mg/topiramate extended release 46 mg ER daily	−9.0 kg		
25 obese women with PCOS	Randomized single-blind, pilot study	16 weeks	placebo	−1.9 ± 1.5 kg	Tongue fat tissue and fat proportion significantly reduced after semaglutide vs. placebo and were assocaited with those in body weight, BMI and WC.	[14]
			semaglutide 1.0 mg	−5.2 ± 4.0 kg		
182 women with PCOS	Randomized controlled trial	12 weeks	metformin 1000 mg BID	−3.6 kg	There was a significant decrease in WC in both treatment groups, and exenatide group was better in changes of WC than metformin group.	[36]
			exenatide 10 μg BID	−5.2 kg		

Legend: WC—waist circumference, VAT—visceral adipose tissue, SAT—subcutaneous adipose tissue.

### 2.2. The Additional Metabolic Effects of GLP-1 Receptor Agonists in PCOS

Beyond its weight loss effect, multiple studies provided additional insight into the metabolic benefits of GLP-1RAs in PCOS. Due to the high prevalence of prediabetes in PCOS, additional insights could be gained from a study in which metformin, exenatide, and their combination were studied to explore their effect on prediabetes remission rate. The remission rate of the combination group (64%) or exenatide group (56%) was significantly higher than that of the metformin group (32%), most likely due to the improvement of postprandial insulin secretion. It is also essential to notice that the effects of exenatide therapy persisted after 12 weeks of drug washout, suggesting possible cellular metabolic treatment legacy effect [37]. Furthermore, Yaribeygi et al. suggested that GLP-1RAs can also improve insulin sensitivity, by proposing eight potential molecular pathways [38].

In PCOS and its common comorbidities, circulating levels of adipose-secreted zinc-α2-glycoprotein (ZAG), an insulin-sensitizing cytokine, are considerably reduced [39,40]. A study on 82 PCOS women demonstrated that 12 weeks of twice a day exenatide 10 mcg or metformin 1000 mg significantly increased ZAG levels in both treatment arms compared to the baseline, without significant differences between the arms [36]. Further information on the metabolic effects was provided in a study evaluating the impact of exenatide on different metabolites in women with PCOS and matched controls [34]. The three-month trial demonstrated that triglycerides, HDL, LDL, total cholesterol, and branched-chain amino acid metabolism were improved following exenatide therapy [34].

Liraglutide administration in five patients with HAIR-AN syndrome, which represents an extreme case of PCOS with metabolic syndrome, resulted in a significant improvement in insulin resistance, adipose tissue amount, hyperandrogenemia, and the menstrual cycle regularity, despite minimal weight loss, therefore, the measured changes could be attributed to liraglutide action per se [41]. Additionally, a small study primarily investigated the liraglutide effect on liver fibrosis biomarkers in PCOS. Procollagen Type 3 amino-terminal peptide, which is a predictor of cirrhosis, was reduced after the intervention [42].

### 2.3. The Effects of GLP-1 RAs on Menstrual Regularity in PCOS

Despite menstrual regularity being an important treatment outcome in PCOS, the effect of GLP-1 levels or treatment with GLP-1RAs remains insufficiently studied. The first study to investigate the impact of GLP-1RA on the menstrual cyclicity randomized 42 oligo-ovulatory and overweight PCOS women to exenatide, metformin, or both. After 24 weeks, a significant improvement in the ovulation rate was demonstrated in all the groups, with the highest rate in the combination group and the lowest in the metformin-only group. Furthermore, the improvement in menstrual regularity was significantly correlated with a reduction in body weight, suggesting weight loss to be the primary driving factor behind the reproductive improvement [27]. A similar correlation between the change in menstrual frequency and BMI was found in a 26-week randomized, placebo-controlled trial that explored the effect of liraglutide 1.8 mg daily on ovarian function in 72 women with PCOS [43]. The bleeding ratio of 0.87 or above (calculated by the number of menstrual bleedings divided by the number of months in the study period) was achieved in 62% of women in the liraglutide group compared with 28% in the placebo group [43]. However, several additional studies with liraglutide in PCOS found unaltered menstrual rate despite reductions in body weight [20,21,29] and insulin resistance [28]. Potential explanations might include small sample sizes, short duration, and the low liraglutide dose [44].

### 2.4. The Effects of GLP-1 Receptor Agonists on Pregnancy Rate in PCOS

There were two studies that addressed pregnancy rates in women with PCOS after an intervention with GLP-1RAs before conception, both reporting better pregnancy outcomes after the GLP-1RA withdrawal [31,32]. The first study included 176 overweight or obese women with PCOS and investigated the natural pregnancy rate in the following 12 weeks after a 12-week treatment with exenatide [32]. The study participants were randomized to receive either exenatide 10 mcg BID or metformin 1000 mg BID for the first 12 weeks, followed by metformin only for the second 12 weeks in which the natural pregnancy rate was tracked. In comparison to the metformin group, the participants receiving exenatide had significantly improved clinical variables after the first 12 weeks, including weight, total percentage of fat, HOMA-IR, and menstrual frequency. The study’s main outcome, the natural pregnancy rate following pre-treatment, was significantly higher in the exenatide group compared to the metformin group (43.6% versus 18.70%, respectively). Although the study was not designed to investigate the underlying mechanisms of this difference in the reproductive outcome, the authors proposed weight loss to most likely be the main contributor to the improved fertility [32]. The second study included 28 obese women with PCOS and explored intervention with low-dose liraglutide (1.2 mg QD) in combination with metformin. The combination of liraglutide and metformin was superior to metformin alone in increasing both the in vitro fertilization and cumulative (including spontaneous conception) pregnancy rates after pre-treatment in patients that were previously resistant to reproductive treatment. The pregnancy rate per embryo transfer was 85.7% in the combination group, compared to 28.6% in the metformin alone group. The cumulative pregnancy rate in 12 months was 69% in the combination compared to 36% in the metformin group. Those results could provide an additional perspective in understanding the direct reproductive effects of GLP-1RAs since both interventions resulted in comparable weight and visceral adipose tissue reductions, indicating other potential mechanisms of action beyond weight loss [31]. In addition, a case report of a 26-year-old infertile and obese PCOS woman reported successful pregnancy following 2-month preconception treatment with exenatide [45].

### 2.5. The Effects of GLP-1 Receptor Agonists on Cardiovascular Outcomes in PCOS

PCOS is known to be linked to adverse cardiovascular risk since insulin resistance is a vital factor in its pathogenesis, importantly leading to several cardiometabolic abnormalities [46]. In comparison to age and BMI-matched healthy controls, women with PCOS have a 30% increased risk of cardiovascular disease [47]. Whether PCOS is associated with subclinical and clinical atherosclerosis, independent of risk factors that commonly accompany the disorder, is unclear [46]. In recent years, cardiovascular outcomes trials have demonstrated that GLP-1RAs can significantly reduce cardiovascular events in individuals with Type 2 diabetes mellitus, however, the majority of available studies with GLP-1RAs in PCOS did not study cardiometabolic endpoints [48,49].

The first study that was designed to assess cardiometabolic endpoints was a 6-month controlled trial, which published its results in 2015. The effect of daily liraglutide 1.8 mg on weight loss and atherothrombosis markers was evaluated in a small group of PCOS women with obesity and controls. Liraglutide treatment was associated with a significant reduction in atherothrombosis markers in both groups, including inflammation, endothelial dysfunction, and clotting [28]. Two years later, the LIPT study (Liraglutide in PCOS on Markers of Vascular Thrombosis) reported effects of the same liraglutide dose in a 26-week study in 72 overweight PCOS women on markers of thromboembolism and cardiovascular disease. The trial demonstrated significant decreases in peak thrombin concentration and increases in time to start of thrombin generation and time to peak thrombin concentration. In addition, there was an improvement in fibrinolytic activity [50]. Additional cardiovascular biomarkers were reported by this research team in this study group a year later. Liraglutide treatment reduced the levels of the cardiovascular risk biomarkers for subclinical cardiovascular disease, midregional-pro-adrenomedullin by 25%, and midregional-pro-atrial natriuretic peptide by 6% (borderline significance) compared with placebo, whereas copeptin levels did not change [51]. The LIPT study also demonstrated reductions in liver fat content, visceral adipose tissue, and the prevalence of non-alcoholic fatty liver disease [23]. Furthermore, in a 4-month study that assessed the effect 16 weeks exenatide intervention on inflammation, endothelial dysfunction, and fibrinolytic activity in 30 overweight/obese women with PCOS, the treatment showed a significant reduction in the cardiovascular risk markers including cellular adhesion molecule 1, p-selectin as well as e-selectin, and an improvement in the C-reactive protein (CRP) [33].

## 3. Comparison of GLP-1 Receptor Agonists to Metformin and the Potential Role of Their Combination

Due to some overlapping treatment targets of metformin and GLP-1RAs in PCOS, it is reasonable to compare their efficacy. Furthermore, the combination of metformin and GLP-1RAs is mechanistically well supported. In preclinical and clinical models, metformin administration resulted in an increase in GLP-1 concentration [52,53]. This combination could, therefore, enhance the therapeutic index of GLP-1RAs and enable the use of a lower GLP-1RAs dose with the benefits of better tolerability.

A 2019 meta-analysis of eight RCTs included 375 patients and compared GLP-1RAs to metformin. The treatment with GLP-1RAs was more effective in improving insulin resistance and lowering BMI, and waist circumference, however, it was also associated with a higher incidence of headache and nausea. Still, there was no difference in the hormone levels, lipid profile, the number of menstrual bleeds, or other adverse effects [54]. The following year, another meta-analysis published results focusing on the weight loss effect of GLP-1RAs individually or combined with metformin in overweight/obese women with PCOS. It showed that GLP-1RAs were associated with a more significant effect compared with metformin-only treatment in eight eligible RCTs [55].

There were three additional meta-analyses that were published in 2021 that compared the therapeutic effect of metformin, GLP-1RAs, or their combination in PCOS with an overlap of the included studies. A meta-analysis that included six RCTs suggested that liraglutide was superior to metformin only in weight loss. Compared to metformin, the combination group had significantly better results in the reduction of weight, waist circumference, BMI, and fasting levels of glucose and insulin. At the same time, the total number of adverse effects was relatively high in the combination group but similar between the metformin and liraglutide groups [56]. Another study group reported similar results in a meta-analysis of 10 RCTs that investigated the therapeutic potential of GLP-1RAs versus metformin in patients with PCOS (five studies with exenatide and five studies with liraglutide). The authors concluded that GLP-1RAs are significantly more effective than metformin in reducing BMI, waist circumference, and insulin resistance in patients with PCOS, however they are more likely to be associated with some adverse reactions as headache and nausea [57]. The third study selected seven RCTs (three studies with exenatide and four with liraglutide) comprised of 464 overweight women with PCOS. A meta-analysis demonstrated that the use of GLP-1RAs resulted in better effects relative to metformin in the reduction of BMI and insulin resistance, however the quality of the evidence is low. Furthermore, the combination therapy demonstrated similar effects on the main outcomes (menstrual frequency, body mass index, total testosterone, HOMA-IR) as GLP-1Ras alone. GLP-1RAs were also found to be connected with lower waist circumference in comparison to metformin. The other primary or secondary outcomes showed no significant differences between the groups. Gastrointestinal adverse effects were not different between either therapies [58].

## 4. Safety Profile and Tolerability of GLP-1 Receptor Agonists in PCOS

GLP-1RAs do not increase the risk of hypoglycemia, exhibit a good safety profile, and are generally well tolerated in PCOS management. The most common adverse effect is mild to moderate gastrointestinal discomfort, which is observed in up to 40% of patients, however, it results in treatment discontinuation in only up to 5% of the study participants [13]. Gradual dose titration is the best strategy to lower the incidence of those reactions and increase the tolerability of the therapy.

Notably, there continues to be a lack of concrete safety data about the use of GLP-1RAs in pregnancy. The FDA and European Medicines Agency classify them as pregnancy class C. Reproduction-aged women should, therefore, be on effective contraception while on therapy and have a washout period before trying to conceive. A single published case report of a 37-year-old patient with PCOS and Type 2 diabetes mellitus described a normal pregnancy and birth of a healthy child after being on GLP-1RA during the first trimester of pregnancy [59].

**Figure 1:**
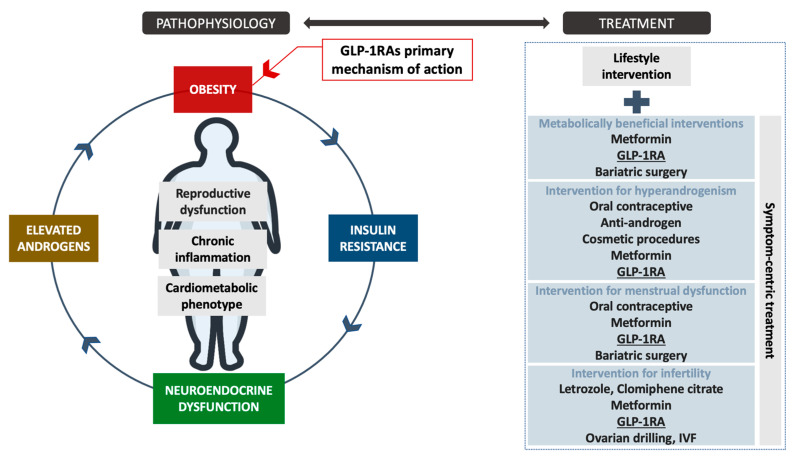
The potential position of GLP-1RAs in the PCOS treatment process and their mechanism of action. The high-level evidence for GLP-1RAs as anti-obesity medications is provided for populations with obesity with or without diabetes. The assessment of PCOS-specific symptom-centric treatment outcomes require future randomized control trials with well-characterized sub-phenotyping of the included cohorts. Legend: GLP-1RAs—Glucagon-like peptide-1 receptor agonists, IVF—In vitro fertilization.

Collectively, the potential position of GLP-1RAs in the treatment approach of various PCOS targets is presented in Figure 1.

## 5. The Potential Benefits of GLP-1 Receptor Agonists in PCOS beyond Weight Management

The key mechanism that is involved in the observed benefits of GLP-1RAs in PCOS is weight reduction. However, some potential additional effects beyond weight loss could hypothetically contribute to some clinical outcomes, yet the evidence from human studies is lacking. The decreased incretin effect is common in several conditions that are accompanied by insulin resistance and particularly well documented in Type 2 diabetes mellitus [60]. Additionally, postprandial GLP-1 levels are decreased in obese individuals [61,62,63,64]. PCOS, commonly characterized by insulin resistance and obesity, was consequently the following hypothesized disorder that was explored for incretin dysregulation. To date, studies of GLP-1 secretion in PCOS are heterogeneous and inconclusive due to small populations, different protocols, and metabolically heterogeneous populations in the relevant studies. Some reported similar fasting and/or postprandial GLP-1 concentrations in PCOS compared with age- and BMI-matched controls [60,65,66,67,68], whereas others reported decreased or increased fasting and/or postprandial concentrations [65,69,70]. Similarly, studies of the association between GLP-1 levels and body weight in PCOS provide inconclusive results [60,65,67,68,69,70]. In most studies, GLP-1 levels were not related to insulin concentration or measurements of insulin resistance.

Obesity is associated with multiple abnormalities in the HPG axis [71,72]; therefore, GLP-1RAs, through their weight loss effect, might indirectly impact the HPG axis. Additionally, GLP-1 most likely has multiple direct effects on the HPG axis [44]. In preclinical models, GLP-1 demonstrates mostly a stimulatory effect on the HPG axis. Therefore, the pharmacological stimulation of GLP-1 receptor (GLP-1R) by the GLP-1RAs might be able to reverse gonadotropins suppression in various states of metabolic imbalance. However, due to the complexity of biological systems, the final effect of GLP-1 on the HPG axis is multifactorial and seems to integrate other synergistic and counterbalanced metabolic and endocrine factors. Furthermore, GLP-1 appears to have a direct antifibrotic and anti-inflammatory effect on peripheral reproductive tissues [44].

GLP-1R is present throughout the HPG axis [44]. GLP-1R mRNA is expressed in the cerebral cortex, hypothalamus, thalamus, and hippocampus [73]. GLP-1 is most likely able to directly modulate the activity of hypothalamic GnRH neurons through multiple potential mechanisms [44,74,75,76,77]. Its appetite suppressing effect is connected to the increase in the electrical activity in the hypothalamic POMC neurons [78]. On the other hand, the expression of GLP-1R on the pituitary gland is significantly lower than in the hypothalamus, therefore, the impact of GLP-1 at the level of the pituitary seems to be predominantly indirect [44,74,75,77]. Additionally, GLP-1R is also expressed in ovaries [79]. GLP-1 was also identified in the follicular fluid in human subjects [80]. In conclusion, the preclinical and clinical studies and the anatomical distribution of GLP-1R suggest that GLP-1 might play a vital role as a modulating signal between the metabolic and reproductive systems [44].

## 6. Future Perspectives

The weight-centric approach with GLP-1RAs offers an opportunity to add a powerful tool to the existing options for PCOS management that provides superior results compared to lifestyle changes or metformin. For this narrative review, a comprehensive literature search was conducted using electronic databases up to 5 May 2022. We predefined the priority of study selection according to the level of evidence (reviews of literature and RCTs over observational studies), on the basis of the population of interest, and the sample size (studies with larger sample sizes were prioritized over case reports). The main conclusions were based on single-center RCTs and one observational study. The possible limitations of our review are related to the heterogeneity between studies with GLP-1RAs in PCOS in settings of considerable variation in the dosing regimen, follow-up duration, population characteristics, effect measurements, and laboratory testing approaches that might all influence the quality of the conclusions of this review. Further well-designed RCTs and cost-effectiveness analyses are needed to support and increase the current level of evidence for the use of GLP-1RAs in PCOS. Larger sample sizes, multi-center, and longer-duration randomized designs will enable the confirmation of their metabolic, reproductive, and cardiovascular risk reduction effects with a proper assessment of the sustainability and safety profile.

Based on the current evidence from the general population, it is recommended to utilize long-acting over short-acting GLP-1RAs, liraglutide and semaglutide over exenatide, because they are specifically approved for the treatment of obesity and have better safety profile and treatment effect. Given that most studies in PCOS used a suboptimal dose of liraglutide for weight management, a high dose of 3 mg daily should be included in future study designs.

Furthermore, semaglutide 2.4 mg demonstrated higher efficacy in body weight reduction and more favorable clinical characteristics compared to all other GLP-1RAs making this long-acting GLP-1RA an advantageous choice [81]. Semaglutide could be the best option also in PCOS, yet robust clinical trials are needed [82]. The oral formulation of semaglutide is also being investigated in 50 adolescent PCOS girls with obesity, currently in recruiting phase. The participants will receive semaglutide (3 or 7 mg tablets once daily) or lifestyle intervention to assess the effects on the hepatic fat and insulin sensitivity, and the estimated study completion date is July 2024 (NCT03919929).

In addition, some neglected outcomes, as health-related quality of life (QoL), should be addressed. Only one cross-sectional study explored the impact of liraglutide treatment on QoL in young and obese women with PCOS compared to age-matched controls. Both groups reported improved QoL, and the improvement was correlated with the amount of weight loss, however, there was no difference between both groups. This potentially suggests that weight loss is the main factor driving enhanced general wellbeing, and successful weight management strategies, including GLP-1RAs, could improve impaired QoL in this population [83].

Based on current evidence, body weight reduction appears to be the primary mechanism of GLP-1RAs action in PCOS. Future studies should also evaluate other tissue-specific effects on GLP-1R, especially at the level of the HPG axis that goes beyond mere weight-lowering potential. An enhanced understanding of the direct impact of GLP-1RAs could help identify PCOS phenotypes that would be the most appropriate treatment candidates.

However, the major concern with the use of GLP-1RAs in PCOS is how to balance the reproductive and metabolic treatment strategies in this population. Both approaches are not mutually exclusive, yet the best choice requires a careful assessment of the clinical context. Knowing a patient’s individual circumstances, precise clinical sub-phenotyping, and regular monitoring are crucial components for the safe and effective use of the new tools. Specific subpopulations of patients with PCOS that might achieve optimal treatment outcomes on therapy with GLP-1RAs need to be properly studied and identified in the future studies. The future designs should also explore the impact of well-defined patients’ characteristics, including sub-phenotyping of the PCOS as well as concomitant metabolic features, patterns of eating behavior, and psycho-social characteristics that might present actionable predictors for enhanced treatment efficacy and help guide future treatment recommendations. Logistical challenges to implement the injectable therapy, titration protocols, and appropriate responses to the potential adverse effects are manageable by the clinical community as soon as we embrace sufficient evidence-based data for this population. We, therefore, need more high-quality research.

## Data Availability

Not applicable.
